# Ageing, *Drosophila melanogaster* and Epigenetics

**DOI:** 10.21315/mjms2020.27.3.2

**Published:** 2020-06-30

**Authors:** Mardani Abdul Halim, Florence Hui Ping Tan, Azali Azlan, Ian Ilham Rasyid, Nurlina Rosli, Shaharum Shamsuddin, Ghows Azzam

**Affiliations:** 1 USM-RIKEN International Centre for Ageing Science (URICAS), Universiti Sains Malaysia, Pulau Pinang, Malaysia; 2 School of Biological Sciences, Universiti Sains Malaysia, Pulau Pinang, Malaysia; 3 School of Health Sciences, Universiti Sains Malaysia, Kubang Kerian, Kelantan, Malaysia

**Keywords:** ageing, longevity, Drosophila, epigenetics

## Abstract

Ageing is a phenomenon where the accumulation of all the stresses that alter the functions of living organisms, halter them from maintaining their physiological balance and eventually lead to death. The emergence of epigenetic tremendously contributed to the knowledge of ageing. Epigenetic changes in cells or tissues like deoxyribonucleic acid (DNA) methylation, modification of histone proteins, transcriptional modification and also the involvement of non-coding DNA has been documented to be associated with ageing. In order to study ageing, scientists have taken advantage of several potential organisms to aid them in their study. *Drosophila melanogaster* has been an essential model in establishing current understanding of the mechanism of ageing as they possess several advantages over other competitors like having homologues to more than 75% of human disease genes, having 50% of *Drosophila* genes are homologues to human genes and most importantly they are genetically amenable. Here, we would like to summarise the extant knowledge about ageing and epigenetic process and the role of *Drosophila* as an ideal model to study epigenetics in association with ageing process.

## Introduction

Ageing is generally defined as progressively getting older and classically coupled with senescence. To date, both phenomena are inevitable for every living organism. The failure of organisms responding to stress encompassing both internal and external factors when they aged will increase the risk of acquiring myriads of diseases. This will eventually lead to death (1). Additionally, external factors such as lifestyle, the quality of the living environment and social environment play essential roles in ageing process (2).

**Figure 1:**
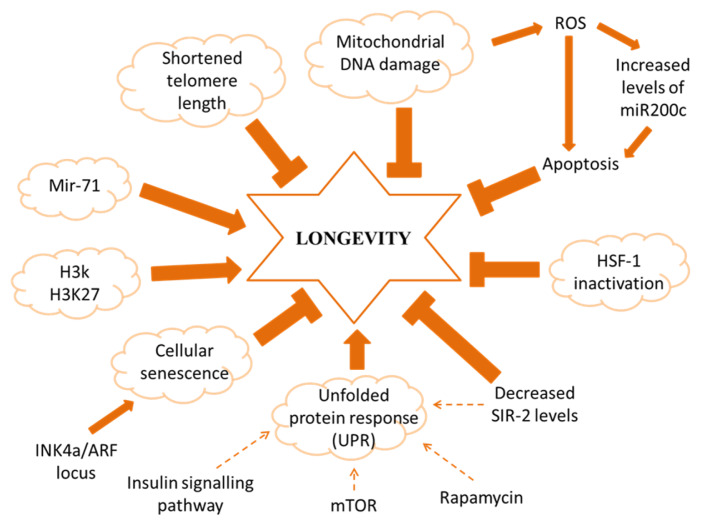
Factors that contribute to longevity. Arrows and T-ended lines indicate activation and repressive interactions respectively, dotted lines show regulatory actions

Currently, there are more than 300 existing ageing theories and they are mostly related to morphology and biochemical changes that arise during ageing (3). Failure in the homeostatic system can disrupt the balance of cell biological system and thus gave birth to the thought of biological ageing as the consequences of homeostatic frameworks incompetency. Another ageing theory suggests that the potential lifespan is controlled by the energetics of all molecules present during and after cell maturation to which it has been tested both theoretically as well as experimentally. Recently, one ageing theory suggested that ageing is an unconstrained thermodynamic process accomplished by all living organism in which it involves an open thermodynamic framework and balancing it with a property of growth (4). Regardless of the variation, all of them possess a common goal; to understand the fundamental of the ageing process. Figure 1 summarised factors that contributed to longevity.

### The Epigenetics of Ageing

Multicellular organisms are composed of many types of differentiated cells—each with distinct functions. During development or cell differentiation, cellular potentials are restricted. In turn, cells acquire functions required for each type of differentiated cells. Large scale changes in gene expression underlie these differentiation processes, with each heritable change in gene expressions are governed by ‘epigenetic’ controls. For epigenetic control, changes in deoxyribonucleic acid (DNA) sequences are not involved. Rather, epigenetic controls utilise genomic modifications such as DNA methylation or post-translational modifications of histone proteins, among others. Currently, some small ribonucleic acids (RNAs) and/or non-coding RNAs are classified as epigenetic regulators. The epigenetic modifications are heritable from one cell generation to the next and epigenetic regulations are operated throughout the entire life span of any organisms to maintain integrity of their life. However, epigenetic modifications are not irreversible; these can be changed in response to environmental stimuli or other factors, e.g. ageing. Numerous studies demonstrated that epigenetic statuses directly or indirectly affect ageing process or vice versa. Ageing is, needless to say, a highly complex phenomenon involving many distinguishable players and different types of age-related epigenetic alterations can be detected depending on genomic domains, cells, tissues or organs examined. Therefore, effects of a particular type of epigenetic modification on ageing process should be vigilantly evaluated. The reduction of DNA methylation, i.e. hypomethylation, that is often observed as organism age is a good case in point. Another specific example is de-repression of IAP retrotransposon in the aged mice due to DNA hypomethylation (5). In contrast, a number of CpG dinucleotides (cytosine nucleotide is followed by a guanine nucleotide in 5’–3’ direction) in CpG islands showed a reproducible increase in DNA methylation observed in aged mice and humans (6) and these DNA methylation changes can be used as epigenetic ‘age predictors’ or ‘ageing clocks’ (7). Therefore, both increase and/or reduction of DNA methylation may be correlated with ageing. In the following paragraphs, further examples of studies focused on epigenetic alterations associated with ageing will be amply described.

### DNA Methylation

Nucleic acid modifications such as DNA methylation is one of the classic examples of epigenetics. Most studies on DNA methylation focused on carbon 5 of cytosine (5mC) in CpG islands. This phenomenon is normally associated with heterochromatin and gene expression in organisms.

On CpG islands, 5mC methylations often occur at gene promoters and typically cause gene silencing (8, 9). Some of the ways that DNA methylation exert such control are by affecting binding of transcription factors and patterning of histone modifications (10, 11). An important example of gene silencing by DNA methylation in humans can be observed among some tumour suppressor genes in cancer cells (12, 13). As ageing progresses, DNA methylation may silence those genes and thus allow unchecked growth of cancer cells. Similar scenarios have also been observed among ageing-related diseases other than cancer, such as Alzheimer’s disease, where significant changes in DNA methylation in brain tissues were found (14, 15). Another study involving cells from mice and humans with progeroid syndromes showed DNA methylation profiles and histone modifications that epitomise those found in normal ageing organisms (16).

Although 5mC is the most extensively studied methylation form in DNA, other methylation mechanisms have also been discovered, namely cytosine methylation at non-CpG site (17) and N6 adenine methylation (6mA) (18). Recent work in *Caenorhabditis elegans* (*C. elegans*) suggested the presence of a putative 6mA DNA methyltransferase (DAMT-1) which is believed to be responsible for 6mA modifications. More interestingly, the study found evidence of high possibility that these 6mA modifications were heritable in *C. elegans*, thus passing the epigenetic information to their subsequent generations (18). Nevertheless, further study is needed to determine the significance of DNA methylation in mammalian ageing process.

### Histone Modification

Histone modification is a post-translational modification of histone proteins including lysine acetylation, arginine and lysine methylation, lysine ubiquitination and sumoylation, and serine and threonine phosphorylation. Such modifications occur mostly within the amino-terminal of the histone protein in which it extended beyond the surface of the nucleosome (19).

Studies in nematodes and flies demonstrated that with the deletion of histone methylation component complex, H3K4 and H3K27, longevity of both organisms can be extended (20). In addition, a study in worm demonstrated that inhibition of histone demethylases, H3K27 can also extend its lifespan. The longevity effects may have been related to circadian rhythm regulation, which was found to involve post-translational modifications of histone. Disruption of circadian clock is significantly associated with ageing-related diseases such as cancer and vascular diseases (21, 22), owing to its importance in regulating a vast number of physiological systems involved in temporally-controlled fluctuations (23). Circadian locomotor output cycles kaput transcription factor (CLOCK) function as an acyltransferase at K9 and K14 of histone H3 and interacts with other histone acetyltransferases (24). A genome-wide chromatin immunoprecipitation study demonstrated that histone H3K4 trimethylation and H3K9/H3K27 acetylation were co-ordinately oscillated at transcription start sites as well as H3K4 monomethylation and H3K27 acetylation at enhancer site (25).

Caloric restriction (CR) and fasting have the capability to improve health as well as extend lifespan in various organisms. Changes in the nutrition of metabolic cues will also extend lifespan and attenuate age-related phenotype in a clock-dependent manner (26). A study in mice revealed de novo oscillating genes under CR display an enrichment of nicotinamide adenine dinucleotide (NAD)-dependent deacetylase sirtuin-1 (SIRT1) in the liver, followed by noticeable circadian hepatic signatures in NAD+- related metabolites and also global protein acetylation (27).

Sirtuin family from adenosine diphosphate (ADP) ribosyltransferase and also NAD-dependent protein deacetylase has been studied widely for their potential in anti-ageing ability. A single sirtuin gene (Sir2) in yeast, flies and worms exhibit an exceptional longevity activity (28). In *Saccharomyces cerevisiae* (*S. cerevisiae*), overexpression of Sir2 will extend their replicative lifespan (29) and in worm (Sir2.1) and fly (dSir2) orthologues, it could extend lifespan for both organisms (29) .

### Transcriptional Modification

Ageing is often linked to transcriptional turbulence and abnormality in the transcribed mRNA resulting in countless age-related transcriptional changes being discussed in various species. During ageing, histone mark H3K36me3 was shown to promote precision in transcription process. A study in *C. elegans* demonstrated with sustained level of H3K36me throughout life will have the less transcriptional modification and also promotes longevity (30). In addition, mianserin-treated *C. elegans* or carrying daf-2 mutation also showing the same traits as histone mark H3K36me3 where it promotes transcriptional precision and increased *C. elegans* lifespan (31).

### Non-Coding RNA in Ageing

Non-coding RNA (ncRNA) is an RNA molecule that does not code or translate into any protein. Although they are not coding for any functional proteins, they play significant roles in many cellular processes including ageing. miRNA LIN-4 is one of the first ncRNA reported to affect ageing process. miRNAs or microRNAs are a class of small, endogenous ncRNAs of 21–25 nucleotides in length that typically exert post-transcriptional gene regulations by targeting and suppressing mRNAs (32). Expression of this ncRNA in *C. elegans* was observed to modulate its lifespan by managing normal temporal control of diverse post-embryonic developmental processes. One of them is by suppressing the expression level of a LIN-14 protein starting in the first larval stage, a protein crucial in developing adult structures such as cuticles and vulva (33). By using the same model organism, miR-71 via the dauer 16/forkhead box O (DAF-16/FOXO) pathway was able to enhance the longevity of *C. elegans* (34). miR-71 is a known repressor for ALG-1/Argonaute. An increase of ALG-1 level in miR-71 mutant will significantly increase the global miRNA expression and eventually lead to dysregulation of mRNA. Under this circumstance, the variability of expressed genes, as well as longevity, will be diminished (35).

In human endothelial cells, an increase in reactive oxygen species (ROS) level was noticed to induce miR-200c and promote apoptosis including senescence (36). Various studies involving human cell lines and mouse model reported the miR-29a were handsomely expressed in neurons of an adult mouse while the expression of miR-29b also increases in various part of mouse central nervous system (37). However, Alzheimer’s and Huntington’s disease mouse and human model showed that the expression of the neuroprotective miR-29 family had declined (38).

### *Drosophila melanogaster* as a Model to Study Ageing

#### History of Drosophila melanogaster in research

*Drosophila melanogaster* ( *D. melanogaster*) or commonly known as fruit fly belonging to the family of Drosophilidae, has been used as a model organism to study genetics and inheritance for more than 100 years. Then, Thomas Hunt Morgan, dubbed the ‘father’ of *Drosophila*, exploited this organism to study heredity and eventually won him the Nobel Prize in Physiology or Medicine in 1933. Later on, his student, Hermann Muller won the Nobel Prize in Physiology or Medicine in 1946 for the study on the effect of x-rays towards genes and chromosome using *Drosophila* as model organism (39). Subsequently in 1995, Christiane Nusslein-Volhard, Eric Wieschaus and Ed Lewis also won the Nobel Prize in Physiology or Medicine for their discoveries on the role of genetic control in the early development of fly embryonic cell (40) and most recently, Jeffrey C. Hall, Michael Rosbash and Michael W. Young went on to win the 2017 Nobel Prize in Physiology or Medicine for their work in elucidating the mechanism involved in controlling circadian rhythm by using *D. melanogaster* as model organism (41).

*D. melanogaster* practically possesses advantages over other model organisms in ageing research. First and foremost, their lifespan is short with a mean life of approximately 60 days to 90 days. Furthermore, they are genetically amenable. The genome of D. melanogaster was sequenced in 2000 which is freely available in ‘Flybase’ database. Deriving from the genome data, approximately 50% of the fly genes having homologues in human (42) with roughly 75% of known human disease genes have fly homologues (43). Various methods have been developed to facilitate in *Drosophila* research specially to generate mutant as well as gene expression system. The most common genetic manipulation used in *Drosophila* is Gal4-UAS and GeneSwitch Gal4 (GSG)-UAS system for gene expression, insertion mutagenesis by P-element and gene knockdown by using RNA interference (RNAi) (44). All these features consolidate *Drosophila*

### Studying Ageing in D. melanogaster

#### Diet restriction

Dietary restriction is one of the most important factors in the ageing study. This regime has been shown to extend the lifespan of mammals (45) and flies (46), among others. A recent study revealed that specific nutrients rather than overall calories mediate longevity to which dietary protein played a significant role in it (47). Moreover, a separate study pinpoints the effect of a single essential amino acid specifically methionine in promoting longevity. This study showed that by limiting the amount of methionine in diet, it can extend the lifespan for both flies and mice. Unfortunately, the effect of longevity is also accompanied by reduced growth rate for mice and infertility in flies (47) .

#### Insulin/IGF-1 like signaling pathway

Insulin is typically the most studied peptide hormones and is crucial in carbohydrate metabolism. Insulin and insulin-like peptide have been discovered in various organisms including flies. In humans, an insulin enzyme and two insulin-like growth factor (IGF) were identified in addition to relaxin and human insulin-like peptides (INSL3–7) (48). In *Drosophila* genome, eight insulin-like peptides (*ilp*1–8), one insulin receptor and the intracellular components P13K were identified (49). Mutant with ilp2 knockdown have shown increased lifespan and this effect is enhanced with the additional knockdown of *ilp*3 and *ilp*5 (50). Surprisingly, the mutants also acquire additional phenotypes like supplemental tolerance to heat, lipophilic toxins and ROS, thus, indicating the involvement of transcription factors (51). The overexpression of FOXO in the gut and fat tissues of D. melanogaster single out 5 transcription factors that could possibly involve in longevity. Among them, one is recognised as the anterior open (AOP), an ETS-family transcriptional repressor. Activation of this AOP also will elevate lifespan in *D. melanogaster* (52).

#### The mechanistic target of rapamycin signaling network

The mechanistic target of rapamycin (mTOR) signaling pathway is an amino acid signaling kinase that plays a central role in cell growth. mTOR is well regarded as the most important amino acid sensor in cells as slight changes in macronutrient dietary response have been closely linked to longevity. Reducing the mTOR function has been shown to extend the lifespan in *C. elegans* (53) and *Drosophila* (54), among others.

Rapamycin is a well-known inhibitor of TOR activity. Feeding rapamycin to flies can overwhelm the life-shortening effect of high protein dietary (55). Such inhibition will trigger autophagy, and autophagy will do its job in dismantling all dysfunctional components of the cell leading to a longer life (56). The overexpression of autophagy components ATG1 (57) via rapamycin treatment also demonstrated longevity in flies.

One of the most frequently studied molecular targets of mTOR is translational repressor 4EBP and translational activator S6 kinase. 4EBP was shown to interact with rapamycin, hence, modulating lifespan (55) while knocked-down S6 kinase literally extend the lifespan in both flies (54) and mice (58). Transcriptional regulators, GATA is responsible in modifying the amino acid-sensitive transcription via TOR-dependent pathway in yeast and mosquitoes (59). The knockdown of this GATA transcription factors in flies gave it the ability to suppress the classical effect of shortened-lifespan due to high amino acid dietary (60).

#### Drosophila gut and muscle

Gut, without a doubt, is one of the most important organs in flies as it facilitates digestion and absorption of nutrients into the body. In addition, the gut serves as a home to microbes which in turn provide necessary protection to the host from ingested pathogens and toxins (61). Interestingly in *Drosophila* gut, few locations in the gut serve as a reservoir for active stem cells, an important component in ageing studies (1). In ageing *Drosophila*, over-proliferation of intestinal stem cells will lead to the development of intestinal epithelium dysplasia (62, 63). *Drosophila* protects their gut against dysplasia or microbial dysbiosis by maintaining their innate immunity (64) and modifying the local inflammatory signaling (65). Hence, protecting flies’ gut from dysplasia can have beneficial effect towards gut microbes and thus enhancing the *Drosophila*’s lifespan.

Structural deterioration of skeletal muscle is an age-related phenomenon in *Drosophila*, and the function will decline in parallel with ageing. Due to lack of stem cells, *Drosophila* do not have the luxury to regenerate their muscles (66). Myoglianin is an important ligand that is indirectly involved in health maintenance in *Drosophila*. The overexpression of myoglianin was shown to enhance climbing ability with age and also increase *Drosophila* longevity (67). On top of that, the overexpression of muscle-specific dFOXO in adult flies was shown to reinforce proteostasis via autophagy mechanism that in turn extend the lifespan of *Drosophila*.

#### Alzheimer’s disease in Drosophila

Alzheimer’s disease (AD) is a progressive neurodegenerative disorder that affects the brain functions and neurons of its patients (68). Dubbed as the most common dementia worldwide, Alzheimer’s disease has an estimated prevalence of 47 million people globally in 2018 with medical costs going up to USD604 billion (69). Though scientists have yet to discover the exact mechanism that governs AD, however, a main hallmark protein has been widely perceived as the culprit to the disease. Amyloid plaques are histopathological lesions that can be found in the brains of AD patients. These plaques are the result of the aggregation of amyloid beta (Aβ), an extracellular by-product of physiological processes from neurons (70).

**Figure 2:**
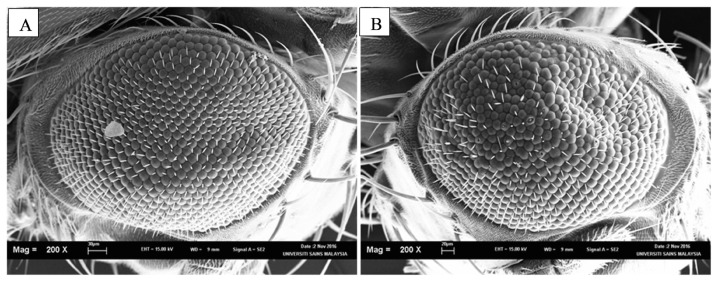
SEM pictures of the *Drosophila* compound eye at magnification 200x. A) An eye of a wild type fly. B) An eye of a transgenic fly expressing Aβ42

*Drosophila* has and continues to aid scientists and doctors in our fight against this disease. Though they do not possess the homolog to the Aβ gene, but they can still express human Aβ gene (71). This is done by taking the advantage of the upstream activator sequence-GAL4 (UAS-GAL4) system. Two lines of *Drosophila* are employed; one carrying a tissue specific promoter fused with the GAL4 gene while the second line contains a UAS gene with the human Aβ gene. In essence, the tissue specific promoter drives the expression of GAL4 proteins which activates UAS gene to produce Aβ peptides. Thus, progenies of both lines will express Aβ in a time and tissue controlled manner (72). For instance, when paired with UAS-Aβ, the eye specific glass multimer reporter-GAL4 (GMR-GAL4) driver results in the distortion of the fly’s compound eye when compared to the eye of a wild-type *Drosophila* (Figures 2A and 2B). The severity of degeneration in the *Drosophila* eye, termed as rough eye phenotype (REP) indicates the toxicity degree of the Aβ peptides. In the *Drosophila* brain, the two most common Aβ peptides formed are Aβ40 and Aβ42. The AD *Drosophila* model has shown that Aβ42 is the more neurotoxic species of the two (73). In addition, the AD *Drosophila* model expressing Aβ42 with the Arctic point mutation which enhances Aβ protofibril formation and intracellular Aβ accumulation exhibited the possibility that Aβ oligomers could also cause neurotoxicity and not just aggregated Aβ (71).

### Drosophila Epigenetics

*D. melanogaster* is a well-known model organism to study genetics as well as epigenetics. Exploration of epigenetic in *Drosophila* led to the unraveling of histone modifications, mobile genetic elements and many more. The epigenetics of *Drosophila* has been comprehensively studied and large number of histone modifications have been identified in different cells and tissues (74).

### Histone Methylation

The methylation of histones was shown to improve lifespan in *Drosophila*. The level of histone methylation is generally dependent on the activity of histone methyltransferase and demethylase. High concentration of heterochromatin protein 1 (HP1) associated with chromatin was shown to slower the senescence effect of *Drosophila* (75). On the other hand, reduction in HP1 methylation resulted in HP1 loss followed by the declining level of heterochromatin and eventually to ageing (75). A recent study reported that reduced level of histone demethylase and methionine metabolism can cause lethal effect to *Drosophila* including a glitch in cell proliferation apart from defect in wing development (76). Changes in expression level of H3K27me have also been documented to produce effect on the *Drosophila*’s lifespan. Heterozygous mutation of the E(z) and Ese subunit of Polycomb repressor complex 2 (PRC2) downregulate H3K27me and thus promotes lifespan extension (10). Likewise, the introduction of H3K4 methyltranferase to the Trithorax complex (trx) increases the level of H3K27me resulting in a reduced lifespan (10). H3K9-demethylase was shown to activate diverse anti-ageing genes including Rpd3, Hsp22, Hk, and also eag. Hypomorphic mutation of this demethylase has been shown to negatively affect longevity in *Drosophila* (77).

### DNA Methylation

DNA methylation is a process in which a methyl group is added to the DNA mediated by methyltransferase. In *Drosophila*, there is only one recognised DNA methyltransferase, Dnmt2, in which it acts upon adenine and cytosine residues (78). In addition, overexpression of Dnmt2 has been shown to control the expression of a few heat-shock proteins in which they are associated with the ageing process (79). Hydroxylation of methylated cytosine residues to form the intermediate product is catalysed by Tet protein. The intermediate product is hypothesised to be associated with demethylation effect leading to DNA relaxation and promote gene derepression (79).

### Remodeling of Nucleosome

Nucleosome is a basic unit of DNA packageing in eukaryote consisting of DNA segment coiled around histones core. In *Drosophila*, there is only one nucleosome-remodeling factor, dMi2 in which is known to involve in ageing (80). Down-regulation of dMi2 causes chromatin to condense (81) and increase lifespan and also acquire more resistance towards oxidative stress (80). Before dMi2 can act upon its target, it must be modified first and that modification is done by poly (ADP-ribose) polymerase (PARP) (82). In addition, PARP also involves in other ageing-related mechanism like DNA repair (83), biogenesis of ribosome (84) and also cell death (85).

### Transposable Elements in Drosophila

Transposable elements are mobile genetic element that constitutes up to 30% of *Drosophila* genome (86). The mobile genetic elements play significant roles in gene regulation in *Drosophila* ageing (87). During normal ageing, several transposable elements were exceptionally active in *Drosophila*. Additionally, the mutation of Argonaute 2 resulted in an intensified expression of the transposon in the brain. Temporal progression of this situation will eventually leads to memory impairment and reduced lifespan (88). On the other hand, reverse transcriptase inhibitors like phosphonoformic acid, lamivudine and also dideoxyinosine were shown to increase longevity in *Drosophila* (89). Small interfering RNA (siRNA) is responsible for the formation of repressive heterochromatin in somatic cells and causes the complete suppression of the activity transposable element (90). Maintaining repressive heterochromatin and silencing the activity of transposable element can promote longevity by easing the DNA damage caused by genetic transposition.

## Conclusion

Cellular health is managed by a series of biological processes. Continuous challenges or stresses to the cells will trigger an internal response in order to curb the stresses at bay. Failing to do so would result in the cells being damaged and eventually, cell death. Fortunately, each organism has their own protection mechanism to overcome such challenges. Studies in model organisms including human have continually established new theories, introducing various approaches to study cell senescence and ageing process. The emergence of epigenetics has contributed enormously to the ageing field. Now, scientists are focusing more on DNA methylation and histone modification towards understanding the fundamental of ageing process. Of course, scientists also have developed various model organisms to aid their studies in ageing. D. melanogaster has been widely used as a model organism and has been critical in establishing the understanding towards the molecular of ageing. Having more than 50% of human gene homologues, while capable in portraying more than 75% of human diseases, low maintenance and most importantly genetically amenable signifies *D. melanogaster* as a perfect model to study ageing (42, 43).
